# Energy-, Cost-, and Resource-Efficient IoT Hazard Detection System with Adaptive Monitoring

**DOI:** 10.3390/s25061761

**Published:** 2025-03-12

**Authors:** Chiang Liang Kok, Jovan Bowen Heng, Yit Yan Koh, Tee Hui Teo

**Affiliations:** 1 College of Engineering, Science and Environment, University of Newcastle, Callaghan, NSW 2308, Australia; jovanbowen.heng@uon.edu.au (J.B.H.);; 2 Engineering Product Development, Science, Mathematics and Technology, Singapore University of Technology and Design, Singapore 487372, Singapore

**Keywords:** IoT, hazard detection, adaptive monitoring, energy efficiency, cost-effective solutions, CNN, ESP32-CAM, real-time alerts

## Abstract

Hazard detection in industrial and public environments is critical for ensuring safety and regulatory compliance. This paper presents an energy-efficient, cost-effective IoT-based hazard detection system utilizing an ESP32-CAM microcontroller integrated with temperature (DHT22) and motion (PIR) sensors. A custom-built convolutional neural network (CNN) deployed on a Flask server enabled real-time classification of hazard signs, including “high voltage”, “radioactive”, “corrosive”, “flammable”, “no hazard”, “no smoking”, and “wear gloves”. The CNN model, optimized for embedded applications, achieves high classification accuracy with an F1 score of 85.9%, ensuring reliable detection in diverse environmental conditions. A key feature of the system is its adaptive monitoring mechanism, which dynamically adjusts image capture frequency based on detected activity, leading to 31–37% energy savings compared to continuous monitoring approaches. This mechanism ensures efficient power usage by minimizing redundant image captures while maintaining real-time responsiveness in high-activity scenarios. Unlike traditional surveillance systems, which rely on high-cost infrastructure, centralized monitoring, and subscription-based alerting mechanisms, the proposed system operates at a total cost of SGD 38.60 (~USD 28.50) per unit and leverages free Telegram notifications for real-time alerts. The system was validated through experimental testing, demonstrating high classification accuracy, energy efficiency, and cost-effectiveness. In this study, a hazard refers to any environmental condition or object that poses a potential safety risk, including electrical hazards, chemical spills, fire outbreaks, and industrial dangers. The proposed system provides a scalable and adaptable solution for hazard detection in resource-constrained environments, such as construction sites, industrial facilities, and remote locations. The proposed approach effectively balances accuracy, real-time responsiveness, and low-power operation, making it suitable for large-scale deployment.

## 1. Introduction

Ensuring safety in hazardous environments such as industrial sites, construction zones, and public infrastructure remains a significant challenge. Global statistics highlight that workplace hazards, including electrical risks, chemical spills, and fire outbreaks, contribute to substantial property damage and loss of life annually [1,2]. Traditional hazard detection systems predominantly rely on fixed surveillance infrastructure, manual inspections, or cloud-based processing, rendering them resource-intensive, costly, and impractical for remote or energy-constrained settings [3,4]. The static operation of these systems, which continuously capture and process data regardless of environmental conditions, results in high energy consumption, increased operational costs, and delayed response, particularly in dynamic environments [5,6].

A critical, yet often overlooked aspect of real-time hazard detection is the freshness of transmitted data. The Age of Information (AoI) metric, which quantifies data timeliness, is crucial in Internet of Things (IoT) applications to ensure up-to-date insights for hazard detection systems [7]. Unlike traditional latency metrics, AoI measures how current the received information is, providing timely and actionable insights [8]. Although this study does not explicitly implement AoI-aware optimization, the adaptive monitoring mechanism inherently enhances data freshness by dynamically adjusting sensing activity based on hazard occurrences.

Specific scenarios—such as remote deployments, temporary field operations, and small-scale enterprises—often encounter significant budget and power constraints, particularly in developing regions or isolated locations, where reliable power sources and high-cost infrastructure are often limited [9,10]. Addressing these challenges requires a flexible, cost-effective, and energy-efficient approach capable of operating efficiently in resource-constrained environments.

This study introduces a novel IoT-based hazard detection system that provides a cost-effective and energy-efficient solution through the integration of several innovative features:

- **Adaptive monitoring mechanisms:** Dynamically adjusts data acquisition rates, leading to significant power consumption reductions, as demonstrated by Zhang et al. [11].
- **Custom-Built CNN Model**: Achieves an F1 score of 85.9%, optimized for real-time embedded deployment [12].
- **Lightweight Flask-Based Server**: Supports real-time hazard classification while minimizing computational and communication overhead [13].
- **Age of Information Implementation**: Maintains data freshness without redundant transmissions [14].
- **Affordable System Design**: Utilizes the ESP32-CAM microcontroller, sensors, and free Telegram notifications for cost-effective deployment [15].
- **Comparative Evaluation**: Demonstrates superior energy efficiency, accuracy, and deployment feasibility against traditional surveillance models [16].

The remainder of this paper is structured as follows. Section 2 reviews the relevant literature on IoT-based hazard detection, adaptive sensing, AoI-aware monitoring, and machine learning-based classification. Section 3 details the system architecture, including hardware components and software design. Section 4 outlines the methodology, covering dataset preparation, model training, and deployment strategies. Section 5 discusses experimental results and performance evaluation, while Section 6 explores the system’s benefits, limitations, and areas for improvement. Finally, Section 7 concludes the paper and outlines future research directions.

## 2. Literature Review

The increasing adoption of Internet of Things technologies has transformed safety and monitoring systems, enabling real-time data gathering, processing, and communication. Traditional hazard detection systems often require extensive infrastructure, entail high costs, and consume significant amounts of energy. These limitations make conventional approaches impractical for resource-constrained environments, particularly in remote or industrial settings.

Recent advancements have led to the development of more efficient and scalable solutions, particularly through the integration of IoT devices with machine learning models [1,2]. This combination enhances system responsiveness, provides data-driven insights, and improves the accuracy of hazard detection, contributing to safer work environments and reduced operational risks.

**IoT-Based Hazard Detection Systems**

The increasing adoption of Internet of Things technologies has revolutionized safety and monitoring systems, providing real-time data gathering, processing, and communication. Traditional hazard detection systems often require significant infrastructure, involve high costs, and demand substantial energy consumption, limiting their applicability in resource-constrained environments. Recent advancements have introduced more scalable and efficient solutions, particularly through the integration of IoT devices with machine learning models [1].

IoT-based hazard detection systems are widely adopted for their ability to offer continuous monitoring, instant alerts, and data-driven insights. Typically, these systems utilize sensor networks to collect environmental data, which are then processed either on-site or via cloud computing. Several studies have confirmed the effectiveness of IoT-based monitoring in both industrial and residential settings. For example, Wan and Wu [4] proposed an advanced IoT-based monitoring system integrating deep learning and edge computing, significantly improving hazard detection accuracy and system responsiveness in industrial safety applications. Similarly, Chen et al. [3] proposed an edge-computing hazard detection system, which significantly reduced latency and power consumption.

Recent advancements have also introduced multi-service IoT deployments, particularly in smart city applications, where integrated sensor networks enhance urban safety and emergency response. Smart city IoT frameworks now incorporate air quality monitoring, flood detection, and predictive infrastructure maintenance, offering a holistic approach to urban hazard management [17]. Integrating hazard detection within a broader IoT ecosystem enables cross-service collaboration, improving scalability and responsiveness in large-scale deployments [18].

**Machine Learning Integration**

Machine learning, particularly convolutional neural networks, plays a critical role in enhancing hazard classification accuracy by extracting complex patterns from visual data. CNNs are widely used in image-based hazard detection due to their robust feature extraction capabilities. However, deploying traditional CNN architectures on low-power IoT edge devices presents challenges related to computational and energy constraints [19].

To address these limitations, lightweight deep learning models optimized for IoT applications have been developed. EfficientNet-eLite, for instance, strikes a balance between accuracy and efficiency, making it ideal for real-time inference on embedded systems [20]. MobileNetV2, which utilizes depthwise separable convolutions, offers a lightweight alternative while maintaining strong hazard detection capabilities [21].

Comparative studies highlight that ResNet-50, VGG16, and MobileNet are among the most widely used architectures, each presenting distinct trade-offs.

- **ResNet-50** excels in learning deep hierarchical features, but its computational demands are higher.
- **VGG16**, known for its high accuracy, requires increased memory capacity.
- **MobileNetV2** and **EfficientNet**, in contrast, are tailored for resource-constrained edge environments, offering optimized performance for real-time IoT deployments [21].

Beyond traditional CNNs, TinyML has emerged as a transformative approach for on-device inference in IoT systems. TinyML enables energy-efficient deep learning on microcontrollers, significantly reducing dependence on cloud computing [16]. By leveraging quantized models and optimized neural architectures, TinyML supports real-time processing with minimal latency, making it a compelling alternative for IoT-based hazard detection [22,23].

**Adaptive Monitoring and Energy Efficiency**

A significant challenge in deploying IoT-based hazard detection systems is managing energy consumption, particularly when devices operate continuously. Adaptive monitoring techniques address this challenge by dynamically adjusting sensing frequency based on real-time environmental conditions. Research has demonstrated that adaptive sensing mechanisms can reduce power usage by up to 40%, significantly extending battery life in low-activity scenarios [5].

A crucial aspect of real-time monitoring is ensuring that transmitted data remain fresh and relevant. The Age of Information metric quantifies data timeliness, ensuring that hazard alerts are based on the most recent observations. Unlike conventional latency measures, AoI evaluates how current the received information is, making it a critical parameter for hazard detection applications [16].

Prior research has explored AoI-aware scheduling techniques to optimize network resource allocation. For instance, optimizing sampling and transmission policies based on AoI constraints has been shown to enhance system responsiveness while reducing redundant transmissions [20]. Although the proposed system does not explicitly implement AoI-based optimization, it inherently maintains data freshness through adaptive monitoring, dynamically adjusting image capture rates based on detected hazards.

Future work could explore AoI-driven scheduling to further enhance update efficiency and optimize power consumption [24]. Adaptive monitoring techniques, such as those demonstrated by the EdgeDRNN framework, highlight the benefits of low-latency edge inference in IoT-based hazard detection systems [25].

**Real-Time Communication and Alert Systems**

Instant communication is essential in hazard detection systems to ensure timely alerts and rapid response. Traditionally, methods such as SMS, push notifications, and messaging platforms have been used for sending hazard alerts. More recently, cost-effective messaging solutions like Telegram have been integrated into IoT-based safety systems, providing scalable and automated alert mechanisms. Martínez et al. [19] demonstrated that using messaging platforms for real-time notifications improves user engagement and ensures rapid information dissemination. Despite its advantages, Telegram-based messaging may face reliability issues in mission-critical applications due to network dependence and platform constraints. As a result, alternative IoT-specific communication protocols such as message queuing telemetry transport (MQTT) and constrained application protocol (CoAP) have been explored.

- **MQTT** is a lightweight messaging protocol designed for low-bandwidth, high-latency environments, making it well suited for hazard alert transmissions [26].
- **CoAP**, on the other hand, is optimized for low-power embedded networks, enabling event-driven messaging in sensor-based hazard detection [27].

By incorporating MQTT and CoAP, IoT-based hazard detection systems can enhance communication reliability, ensuring that critical alerts are delivered even under network constraints. Future implementations could explore hybrid approaches combining Telegram for real-time notifications with MQTT/CoAP for system redundancy in smart city hazard detection frameworks.

**Positioning of the Proposed System**

The system suggested in this study improves upon current IoT-based hazard detection solutions by combining adaptive monitoring with machine learning to enhance energy efficiency and resource management. In contrast to traditional systems that depend on constant data delivery, this setup uses an ESP32-CAM module, an affordable microcontroller capable of capturing and sending images for instant processing on a Flask server. By implementing adaptive monitoring mechanisms, the system can change image capture frequency based on environmental conditions to save power while maintaining performance. In addition, employing free Telegram services for instant alerts guarantees affordable and dependable communication, thus rendering the system appropriate for use in settings with limited resources.

**Age of Information in Real-Time Hazard Detection**

Real-time monitoring systems must not only provide accurate hazard detection but also ensure that transmitted data remain fresh and relevant. The concept of Age of Information is widely used to measure data timeliness in real-time IoT applications, quantifying the time elapsed since the most recent update was received [13]. This metric is particularly critical for hazard detection systems, where outdated information can lead to delayed responses and increased safety risks.

Previous studies, such as “Throughput maximization with an AoI constraint in energy harvesting D2D-enabled cellular networks” [14], have investigated AoI-aware scheduling techniques to optimize information freshness in constrained environments. These techniques often involve optimizing sampling and transmission policies to enhance system responsiveness while reducing redundant transmissions.

Although the proposed system does not explicitly incorporate AoI-based optimization, it inherently addresses data freshness through adaptive monitoring, dynamically adjusting sensing activity based on detected hazards. This adaptive approach ensures that hazard alerts are based on the most recent observations, maintaining data relevance without excessive power consumption.

Future enhancements could explore AoI-driven scheduling to further refine update frequency, minimize redundant transmissions, and optimize network reliability for hazard notifications [15].

Recent advancements in AoI optimization and energy efficiency for IoT systems have demonstrated significant potential. A study by Qin and Ye [28], titled “Age of Information joint optimization for an energy harvesting network with erasure channel” (*IEEE Access*, 2023), proposed a joint optimization strategy for energy harvesting IoT scenarios, focusing on minimizing AoI while considering energy constraints and unreliable channels. Their MSRA-TD3 approach dynamically optimizes data transmission policies, ensuring minimal AoI while maintaining efficient energy usage. This work is particularly relevant to the proposed hazard detection system, as it highlights the benefits of adaptive monitoring mechanisms that prioritize timely data transmission without excessive power consumption. While the proposed system does not explicitly implement advanced AoI optimization, its adaptive monitoring approach aligns with similar principles by adjusting image capture frequency based on hazard activity, contributing to improved energy efficiency and data freshness.

Integrating such optimization techniques could be a potential avenue for future enhancement, offering more granular control over sensing and communication strategies in hazard detection applications.

**Integrated Multi-Service Systems for Hazard Detection**

Modern IoT-based hazard detection systems increasingly adopt integrated multi-service architectures, enabling seamless interoperability across diverse applications, including environmental monitoring, industrial safety, and smart city infrastructure, as demonstrated by Cao et al. [29]. Multi-service systems leverage hierarchical, collaborative, and reconfigurable wireless sensor networks (WSNs) to enhance scalability and efficiency.

For example, research on collaborative sensor networks has demonstrated that hierarchical data fusion can significantly reduce latency and energy consumption while improving real-time decision-making [16]. While the proposed system primarily focuses on hazard detection, its modular design allows for potential integration into multi-service frameworks. Future implementations could explore interoperability with other IoT-based monitoring applications, such as air quality assessment, predictive maintenance, and smart city infrastructure monitoring [30].

**Smart City Connectivity and Dependability**

Reliable connectivity is a fundamental requirement for IoT-based hazard detection in smart city environments, where data transmission delays or failures can lead to critical safety risks [31]. The quality of connectivity (QoC) plays a vital role in ensuring real-time responsiveness in large-scale IoT deployments.

Several studies have explored low-latency communication strategies, such as MQTT-based messaging and edge-assisted real-time processing, to mitigate network congestion and data loss [32]. Additionally, emerging research on wireless edge computing has demonstrated its potential in balancing computational loads while preserving low-latency decision-making for hazard detection systems [33]. Large language models (LLMs) are increasingly being integrated into smart city frameworks to enhance data processing and decision-making, contributing to improved hazard detection capabilities [34].

The proposed system relies on Wi-Fi connectivity for real-time hazard alerts via Telegram, making it suitable for deployments within existing infrastructure. However, network constraints in remote environments may impact reliability. Future enhancements could explore alternative communication protocols, such as LoRa-based messaging or MQTT integration, to improve system robustness in smart city deployments.

**TinyML as an Alternative for Energy-Efficient Inference**

While deep learning models like CNNs deliver high accuracy, they often require significant computational resources and power.

A promising alternative is TinyML, a lightweight machine learning framework tailored for low-power embedded devices. TinyML enables on-device inference with minimal latency, making it an attractive option for IoT-based hazard detection [23]. Research on TinyML-based CNN compression has demonstrated that optimized deep learning models can operate efficiently on ESP32 and ARM Cortex-M processors, achieving competitive accuracy with drastically lower power usage [35].

The proposed system currently offloads CNN inference to a Flask server, allowing for higher model complexity. However, future iterations could explore TinyML-based implementations, enabling on-device processing directly on the ESP32-CAM. Recent studies have explored the use of tensor processing units to enhance energy efficiency in AI applications, demonstrating the potential for substantial performance gains in edge computing scenarios [36]. Such an approach would reduce inference latency, improve power efficiency, and enhance real-time decision-making, particularly in network-limited environments [37].

## 3. System Design and Benefits

The suggested hazard detection system based on IoT was created to offer an affordable, low-energy, and flexible solution for monitoring dangerous situations in real time. The system combines various important parts such as the ESP32-CAM microcontroller, DHT22 temperature sensor, PIR motion sensor, and Flask server for data processing. The structure was created to maximize resource utilization while guaranteeing consistent performance in various settings.

**System Architecture**

**Figure 1:**
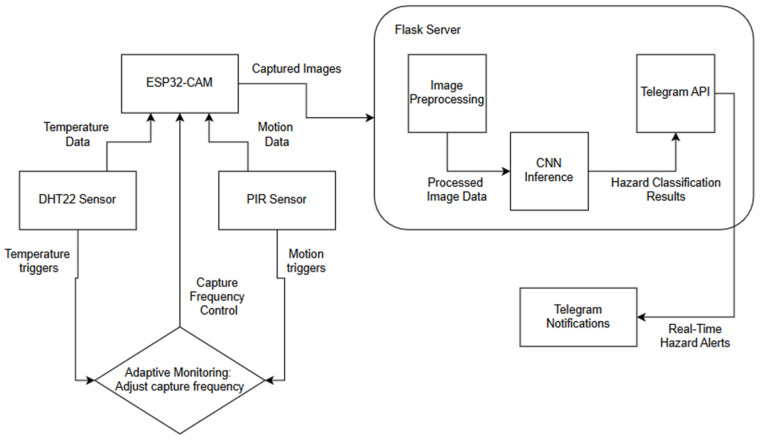
System architecture of the proposed hazard detection system.

The proposed hazard detection system consists of multiple interconnected components designed to enable real-time monitoring, hazard classification, and adaptive sensing. Figure 1 illustrates the system architecture, which includes the following key elements.

1. **ESP32-CAM with Integrated Sensors:** The system utilizes an ESP32-CAM microcontroller for image capture and environmental monitoring. A DHT22 temperature sensor records temperature variations, while a PIR motion sensor detects movement patterns. These sensors provide contextual awareness, allowing the system to dynamically adjust image capture rates based on real-time environmental changes.
2. **Flask-Based Server for Real-Time Processing:** The captured images and sensor data are transmitted via Wi-Fi to a Flask-based backend server. Flask was selected due to its low resource consumption, ease of deployment, and real-time processing capabilities. The server handles image preprocessing and forwards the processed data to a custom CNN model for classification. Performance analysis of TPUs in data center environments highlights the advantages of hardware acceleration for AI applications, offering insights into potential future enhancements of the proposed system [22]. While the lightweight Flask-based server enhances processing speed and reduces network dependence, it also introduces a potential single-point failure risk. To mitigate this, future iterations of the system could incorporate redundancy measures, such as using a backup server or implementing a failover mechanism. This would involve automatically rerouting data to a secondary server if the primary Flask server experiences a failure, enhancing system robustness and ensuring uninterrupted hazard detection in critical environments.
3. **Convolutional Neural Network Model for Hazard Classification:** The CNN was trained on a custom dataset comprising various hazard types. It categorizes images into predefined hazard classes, such as fire hazards, chemical spills, electrical hazards, etc. The model was optimized to balance classification accuracy (F1 score: 85.9%) and computational efficiency, making it suitable for IoT deployment.
4. **Adaptive Monitoring Mechanism for Power Optimization:** The system adjusts its sensing and transmission rates dynamically, reducing unnecessary data transmissions. Example: If motion is detected, the image capture frequency increases; otherwise, it remains in a low-power mode. This mechanism improves power efficiency by up to 37%, extending battery life in remote deployments.
5. **Hazard Alert System via Telegram Messaging:** Once classified, hazard alerts are sent via Telegram in real time to notify users. While Telegram is used for notifications, future iterations could integrate MQTT or CoAP for more reliable, low-latency alert transmission.

By integrating these components, the system, as shown in Figure 1 below, effectively reduces energy consumption and operational costs, making it suitable for resource-constrained environments such as industrial sites, agricultural fields, and remote areas. The architecture’s modular design also allows for future enhancements, including alternative messaging protocols (MQTT, LoRa) and TinyML-based on-device inference. While hierarchical and reconfigurable WSNs offer competitive energy efficiency, they often involve complex network configurations and higher initial costs associated with sensor coordination and network reconfiguration [38,39].

Recent advancements, such as the divisive hierarchical clustering approach for UAV-assisted WSANs, have demonstrated improvements in energy savings and latency reduction, enhancing the feasibility of such systems in dynamic environments [1]. Additionally, hybrid intrusion detection models using federated learning provide robust solutions for managing network security while maintaining energy efficiency in hierarchical WSNs [39].

The proposed system simplifies deployment by using adaptive monitoring and off-the-shelf components, reducing both operational costs and power requirements, making it a viable option for budget-conscious applications. The proposed system uses a local Flask server to process hazard detection tasks, providing real-time analysis and reducing latency compared to cloud-based systems. However, as a single processing point, the Flask server may introduce a potential failure risk in cases of hardware or network disruptions. Although the current implementation does not include redundancy mechanisms, future iterations of the system could integrate failover strategies, such as backup servers or edge computing solutions, to improve reliability in critical deployments.

**Adaptive Monitoring Mechanism**

The proposed system’s adaptive monitoring mechanism is one of its most notable features. Conventional hazard detection systems usually run at a consistent rate, resulting in unneeded energy usage. On the other hand, this system changes the image capture speed depending on the activity identified by the PIR sensor and temperature data from the DHT22.

- **Low-Activity Periods:** When minimal motion is detected, the system reduces image capture frequency, conserving power.
- **High-Activity Periods:** If frequent motion is detected, the system increases the capture frequency to ensure no potential hazard goes unnoticed. This adaptive approach significantly enhances energy efficiency, particularly in environments where activity levels fluctuate throughout the day.

**Data Processing and Classification**

Images that are captured are sent to the Flask server, where a CNN model analyzes them to detect potential dangers. The model was trained on a dataset containing different hazard classes, allowing it to categorize images as “high voltage,” “flammable,” “radioactive,” “corrosive,” “no smoking,” and “wear gloves.” The server records these classifications and is able to send alerts based on certain criteria. If the temperature sensor detects high heat levels and the captured image shows a flammable symbol, a notification is quickly sent through the Telegram bot, enabling fast reactions.

**Communication and Real-Time Alerts**

**Figure 2:**
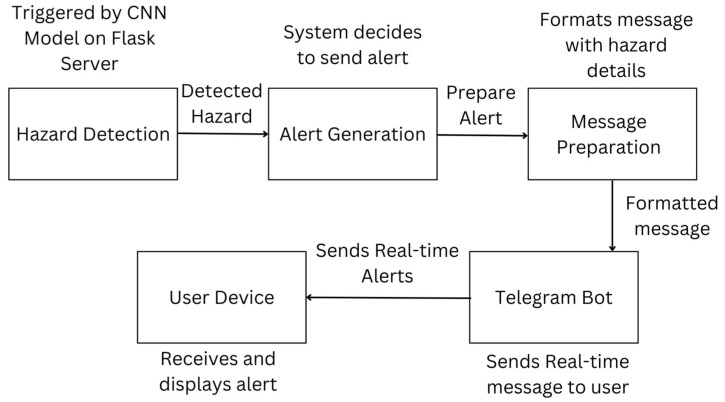
Example of real-time alerts via Telegram demonstrates a sample notification showing a detected hazard and corresponding action.

The proposed hazard detection system integrates a Telegram-based notification system, shown in Figure 2, to provide real-time alerts upon detecting potential hazards. This approach ensures instant notifications without additional service costs, leveraging the Telegram API for automated messaging. The system effectively sends hazard alerts to designated users, enabling rapid response and preventive actions.

**Advantages of Telegram for Real-Time Alerts:**

- **Cost-Effective:** Unlike SMS-based alerts, which incur per-message fees, Telegram messages are free of charge, reducing long-term operational expenses.
- **Scalability:** Telegram supports multi-user notifications, making it ideal for environments where multiple personnel must receive hazard alerts simultaneously.
- **API Integration:** The Telegram API allows for automated hazard notifications, seamlessly integrating with the Flask-based server to provide instant updates.

**Limitations of Telegram as an Alerting Mechanism:**

Despite its advantages, Telegram has some limitations when deployed in mission-critical hazard detection applications.

- **Dependence on Internet Connectivity:** Telegram requires a stable internet connection, which may be unreliable in remote or industrial environments.
- **No Guaranteed Delivery Timeframe:** Unlike SMS-based alerts, Telegram messages are relayed through cloud servers, introducing potential latency issues in high-traffic conditions.
- **API Rate Limits:** High-frequency hazard alerts may be throttled by Telegram’s API restrictions, affecting large-scale deployments.

**Alternative Messaging Solutions for Enhanced Reliability:**

To overcome Telegram’s limitations, the system can be expanded to support alternative communication protocols, including the following.

- **SMS-Based Alerts (Twilio, Nexmo, or AWS SNS):** Offer high reliability with direct message delivery, suitable for industrial safety applications.
- **MQTT:** Enables low-bandwidth, real-time messaging, making it an effective alternative for IoT-based hazard monitoring.
- **LoRaWAN (long-range wide-area network):** Provides long-range, low-power communication, ensuring hazard notifications reach users even in areas with poor cellular connectivity.

The modular nature of the proposed system allows for seamless integration of multiple alerting mechanisms, ensuring that hazard notifications remain reliable and accessible across different network conditions. Future work will explore multi-channel alerting leveraging a combination of Telegram, SMS, and MQTT to enhance system robustness.

**Benefits of the Proposed System**

The design and functionality of this IoT-based hazard detection system offer several key benefits.

1. **Cost-Effectiveness:** The cost-effectiveness of the proposed hazard detection system is validated by comparing its setup and operational expenses with traditional surveillance and monitoring solutions. Conventional systems rely on high-end surveillance cameras, dedicated control centers, and paid alert mechanisms (e.g., SMS or cloud-based notifications), leading to significant long-term costs. The proposed system minimizes costs by:
  - Using low-cost hardware components such as ESP32-CAM, DHT22 sensor, and PIR motion sensors, resulting in a total cost of SG 38.60 per unit compared to USD 500–1000+ for traditional surveillance setups (Table 1).
  - Eliminating subscription-based alerts by utilizing free Telegram notifications instead of paid SMS services, which typically cost USD 35–155 per month [40].
  - Reducing infrastructure costs by running hazard classification on a lightweight Flask server instead of a cloud-based solution, which can incur additional storage and processing fees [41].
2. **Energy Efficiency:** The adaptive monitoring mechanism minimizes power consumption by dynamically adjusting operations based on real-time activity detected by the sensors. During periods of low activity, the system reduces the frequency of image captures, conserving battery life and extending operational time. This energy-saving feature leads to:
  - 31–37% energy savings compared to continuous surveillance models, which consume significantly more power due to 24/7 streaming and data transmission [42].
  - Optimized power usage, making the system suitable for battery-powered and solar-powered deployments, where energy resources are limited.
  - Lower data transmission costs, since images are only sent when a hazard is detected, reducing unnecessary network usage.
3. **Scalability and Flexibility:** The modular design allows for easy integration of additional sensors and components, enabling customization based on specific monitoring needs. For instance, the system can be expanded to include gas sensors, humidity sensors, or even integrate LoRa communication for long-range data transmission. The flexibility of the design ensures that it can adapt to various use cases, from small-scale home monitoring to larger industrial safety networks.
4. **Ease of Use and Deployment:** The system’s reliance on existing free communication platforms (Telegram) streamlines real-time alerting, eliminating the need for complex infrastructure setups. The Telegram bot is easy to configure, and users can quickly set up alerts, modify notification preferences, and monitor conditions remotely, making the system accessible to non-technical users.
5. **Reliability and Real-Time Performance:** By leveraging a locally hosted CNN model on the Flask server, the system delivers prompt processing of captured data and swift alert responses, which is crucial for timely interventions. The local processing ensures that the system does not rely on external cloud services, reducing latency and providing immediate feedback to users.
6. **Instantaneous Communication:** Telegram’s messaging platform ensures that alerts are delivered instantly to the user’s device, allowing for prompt action. The system can notify multiple users simultaneously, making it ideal for scenarios where different personnel must be informed at the same time, such as factory safety managers or emergency response teams.
7. **Cross-Platform Compatibility:** Users can receive alerts on multiple devices, including smartphones, tablets, and desktops, regardless of the operating system (iOS, Android, Windows, etc.). This flexibility makes the system more accessible, allowing users to monitor conditions remotely even if they switch devices, ensuring consistent real-time updates.
8. **Enhanced Security:** Telegram provides secure end-to-end encryption, ensuring that the communication between the system and the user remains private and tamper-proof. This is particularly important for systems dealing with sensitive environments, where information security is crucial.
9. **Customizable Notifications:** The system can be programmed to send tailored messages, including details like the type of hazard, location, time, and recommended actions. Customizing the message format ensures that users receive all necessary information in a clear, concise way, helping them respond more effectively. For example, if the system detects a “flammable” hazard, the alert could include specific instructions to evacuate or take safety measures.
10. **Low Data Usage:** Sending messages via Telegram consumes minimal data, making it efficient for deployment in areas with limited or slow internet connections. This ensures that alerts are not delayed due to connectivity issues, maintaining reliable performance even under constrained network conditions.
11. **Scalability for Large Networks:** The system’s design allows for multiple users to be added to receive notifications, making it suitable for larger teams or organizations that need to monitor multiple sites or areas. This feature is beneficial in industrial settings where several managers or operators might need to be informed simultaneously.
12. **Remote Monitoring and Management**: The ability to receive alerts and monitor conditions remotely makes the system suitable for deployments in hard-to-reach or hazardous areas. This reduces the need for physical inspections, improving safety for personnel and reducing operational costs.
13. **Adaptable for Diverse Environments:** The system is designed to function effectively in a wide range of settings, from residential properties to industrial facilities. Its adaptability means that it can be deployed in warehouses, factories, farms, and construction sites, providing continuous monitoring and real-time feedback to address various safety concerns.
14. **Seamless Integration with Existing Infrastructure:** The system can be easily integrated with existing IoT frameworks, allowing it to complement other monitoring devices and systems. This makes it a versatile addition to current setups without requiring extensive modifications, enhancing overall monitoring capabilities.
15. **Resource Optimization:** By adjusting its operation based on sensor inputs, the system optimizes resource usage, including bandwidth, power, and processing capabilities. This optimization leads to a more efficient system that balances performance and resource consumption, ideal for environments where resources are limited or expensive.

## 4. Methodology

### 4.1. Data Collection and Preparation

The initial step in developing the hazard detection system involved gathering a comprehensive dataset of images for training the machine learning model. The data collection process was automated using a Python V6.78 script that utilizes the bing_image_downloader library. This script allows for the efficient downloading of images based on predefined search terms, such as “flammable sign,” “no smoking,” or “radioactive hazard,” and stores them in a structured directory for easy access.

**Figure 3:**
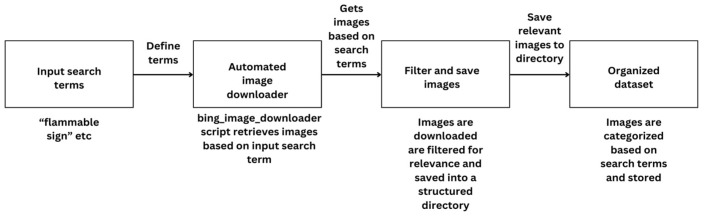
Flowchart illustrating the automated data gathering process for hazard sign images.

The automated nature of this process ensures that a diverse dataset is gathered quickly, which is essential for training a robust model. Each image is downloaded and saved with consistent naming conventions, simplifying further preprocessing. Additionally, the script is designed to handle connection issues by retrying failed downloads, making it reliable for large-scale data gathering [43]. Figure 3 below illustrates the flowchart of the automated data gathering process, showing how images are sourced based on search terms and organized systematically.

### 4.2. Data Splitting

After gathering the images, the dataset was split into training, validation, and testing sets using the splitfolders library. This library makes it easier to replicate the division of data by offering a set seed value, guaranteeing the use of the same data split in various experiments for accuracy. The selected division ratio of 70% for training, 15% for validation, and 15% for testing was chosen to ensure enough data for model training and unbiased evaluation. The automation in the code guarantees that each new run follows this division, enabling consistent monitoring of performance in different setups and repetitions. Proper data partitioning is crucial in machine learning to prevent data leakage, which can lead to inflated performance metrics. Recent studies have highlighted the effectiveness of subject-independent data partitioning strategies in avoiding over-optimistic model performance by ensuring strict separation of training and testing datasets [44].

### 4.3. Data Preprocessing and Augmentation

The raw images collected during data gathering required preprocessing to standardize inputs for the CNN model. Preprocessing steps included resizing images to 150 × 150 pixels and normalizing pixel values to a [0, 1] range. Normalization helps accelerate the training process by ensuring that all inputs are on a similar scale, which is critical for models that rely on gradient-based optimization methods.

The normalization process can be described by the following equation:

(1) $$X_{norm}=\frac{X-\mu }{\sigma }$$

where $X_{norm}$ is the normalized pixel value, *X* is the original pixel value, μ is the mean of the pixel values, and σ is the standard deviation of the pixel values.

In addition to preprocessing, data augmentation was applied to increase the diversity of the training set artificially. During the data preprocessing phase, several challenges were encountered, such as variations in image quality and inconsistencies in lighting conditions.

To address these issues, data augmentation techniques like brightness adjustments, random rotations, and cropping were applied, allowing the model to learn from a diverse set of images. This approach helped improve the model’s robustness, enabling it to generalize better to new and varied data. Techniques such as horizontal flipping, rotation, random cropping, and color jittering were implemented using the torchvision.transforms library.

**Figure 4:**
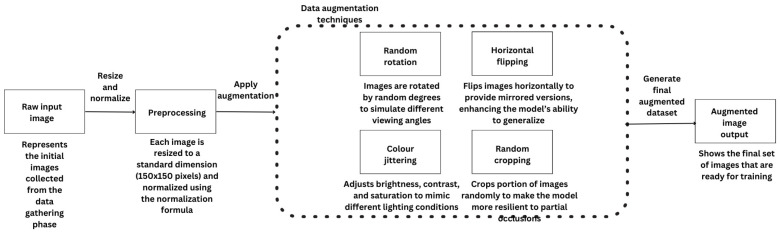
Pipeline illustrating data preprocessing and augmentation techniques used to improve model training.

These transformations were chosen to mimic real-world variations that might be encountered in different environments, such as changes in lighting, perspective, and orientation. For example, color jittering allowed the model to learn to recognize hazards under varying light conditions, while random cropping helped it focus on relevant portions of images even when parts of the sign were partially obscured [43]. Figure 4 below shows the data preprocessing and augmentation pipeline, highlighting each step from initial image input to the final processed output.

### 4.4. Convolutional Neural Network Model Training

The key element of the hazard detection system is a specially made convolutional neural network that was created to categorize images into seven different groups, like “high voltage,” “radioactive,” and “flammable.”

The CNN contains convolutional layers for extracting important features, pooling layers for decreasing spatial dimensions, and fully connected layers for the ultimate classification. In order to avoid overfitting, dropout layers are utilized to randomly deactivate neurons in each training iteration, ensuring the model learns a variety of features without depending on particular patterns. Overfitting happens when the model memorizes patterns from the training data that do not apply well to new, unseen data.

Dropout assists in addressing this issue by prompting the model to acquire a broader range of features. Throughout the training phase, groups of preprocessed images are entered into the network, and the cross-entropy loss function is employed to update the weights. This loss function plays a crucial role in overseeing the training procedure by evaluating the similarity between the predicted probabilities and the true labels. The model’s ability to accurately detect dangers is assessed using metrics like accuracy, precision, recall, and F1 score, giving a complete summary of its performance.

The Adam optimizer was selected for its adaptive learning rate capabilities, facilitating efficient convergence during training. Recent advancements have demonstrated how dynamic parameter adjustment in Adam can improve convergence speed and model generalization in deep learning frameworks [44]. The Adam optimizer combines the benefits of adaptive learning rates and momentum, helping the model converge faster and navigate complex error surfaces efficiently. During the training process, a learning rate schedule was applied, starting at 0.0003 and gradually reducing it at specific epochs. This adjustment allowed the model to fine-tune its weights and improve performance as training progressed.

The cross-entropy loss function used during training is defined by:

(2) $$L=-\frac{1}{N}\sum_{i=1}^{N}\sum_{j=1}^{C}y_{ij}\log\left(p_{ij}\right)$$

where *L* is the cross-entropy loss, *N* is the number of samples, *C* is the total number of classes, $y_{ij}$ is the binary indicator that shows 1 if the class label is correct and 0 if it is wrong, and $p_{ij}$ is the predicted probability that sample *i* belongs to class *j*.

This equation measures how close the predicted probabilities are to the actual labels. Lower values of L indicate better model performance, guiding the training process to adjust the weights in a way that reduces the error.

The cross-entropy loss function was employed for the following reasons.

- **Optimized for Multi-Class Classification:** Unlike MSE, which treats class probabilities as continuous values, cross-entropy directly measures the distance between predicted probabilities and actual labels, making it more effective for classification.
- **Ideal for Softmax Activation:** CNNs use a softmax layer in the final stage to generate class probabilities. Cross-entropy is mathematically suited for softmax, as it ensures stable gradient updates and prevents probability distortions.
- **Prevents Vanishing Gradients:** In deep networks, MSE tends to produce small gradients, leading to slow convergence. Cross-entropy provides larger gradient updates for incorrect classifications, improving training efficiency.
- **Better Probability Calibration:** Cross-entropy ensures that predicted probabilities reflect confidence in classification, making it highly suitable for real-time hazard detection.
- **Computational Efficiency:** The function generates well-structured gradients, which allows faster convergence and more effective weight updates during backpropagation.

Comparison with alternative loss functions:

- **Mean Squared Error (MSE):** MSE is mainly used for regression and performs poorly in classification tasks because it does not account for probability distributions.
- **Hinge Loss:** Hinge loss is effective for binary classification using support vector machines (SVMs), but does not generalize well for multi-class classification.
- **Binary vs. Categorical Cross-Entropy:** Since the proposed CNN predicts multiple hazard classes, categorical cross-entropy is used instead of binary cross-entropy, which is only applicable for two-class problems

Given that hazard classification is inherently a multi-class classification problem, cross-entropy loss is the most suitable choice for maximizing classification accuracy while ensuring efficient training in CNN-based models.

To evaluate the performance of the model, the following metrics were used.

The precision metric is defined by:

(3) $$Precision=\frac{True\;positives}{True\;positives+False\;positives}$$

where true positives are the correct identification instances of a hazard and false positives are the incorrectly identified instances where the model predicted a hazard, but none was present. Precision measures the proportion of correctly identified positive cases out of all the instances the model predicted as positive. High precision indicates a low rate of false alarms.

The recall metric is defined by:

(4) $$Recall=\frac{True\;positives\;}{True\;positives+False\;negatives}$$

where false negatives are instances where a hazard was present, but not detected by the model. Recall indicates the model’s ability to identify all actual positive cases. High recall means the system successfully detects most of the hazards without missing them.

The F1 score metric is defined by:

(5) $$F1\;score=2\times \frac{Precision\times Recall}{Precision+Recall}$$

The F1 score is the harmonic mean of precision and recall, providing a single metric that balances both. It is especially useful in scenarios where there is an imbalance between classes or where both false positives and false negatives need to be minimized.

A high F1 score indicates that the model performs well across both precision and recall, providing a balanced measure of accuracy.

The proposed CNN architecture is specifically designed to balance high accuracy and computational efficiency, making it suitable for ESP32-CAM deployment. The network consists of four convolutional layers, each optimized for progressively deeper feature extraction, followed by a fully connected output layer for multi-class classification.

- **Input Layer (150 × 150 × 3 RGB image):** The model takes RGB hazard sign images of size 150 × 150 pixels, ensuring a balance between resolution and computational cost.
- **Conv Layer 1 (32 filters, 3 × 3 kernel, stride 1, ReLU activation):** Captures low-level spatial features, such as edges and simple textures, to build foundational feature representations.
- **Batch Normalization 1 and Max-Pooling (2 × 2, stride 2):** Improves training stability and reduces spatial dimensions while retaining critical details.
- **Conv Layer 2 (64 filters, 3 × 3 kernel, stride 1, ReLU activation):** Extracts mid-level features, such as curves and distinct hazard sign shapes.
- **Batch Normalization 2 and Max-Pooling (2 × 2, stride 2):** Further compresses feature maps while preserving key information for classification.
- **Conv Layer 3 (128 filters, 3 × 3 kernel, stride 1, ReLU activation):** Detects more complex patterns, including hazard-specific textures and symbols.
- **Batch Normalization 3 and Max-Pooling (2 × 2, stride 2):** Prevents overfitting while enhancing computational efficiency.
- **Conv Layer 4 (256 filters, 3 × 3 kernel, stride 1, ReLU activation):** Learns high-level abstract representations, enabling fine-grained hazard classification.
- **Dropout (35% rate):** Prevents overfitting by randomly deactivating 35% of neurons during training, improving model generalization.
- **Flatten Layer:** Converts the 3D feature maps into a 1D vector, preparing data for the dense classification layers.
- **Fully Connected Layer (neurons = based on extracted features, ReLU activation):** Learns a final feature representation to classify hazard categories.
- **Softmax Output Layer (seven neurons, one per class):** Produces a probability distribution over seven hazard sign categories.

This structure was chosen for the following reasons.

- **Optimized for ESP32-CAM:** The CNN is designed to minimize FLOPs while maintaining classification accuracy, making it efficient for real-time deployment.
- **Progressive Feature Extraction:** The increasing filter sizes (32→64→128→256) allow hierarchical feature learning, improving classification accuracy.
- **Dropout and Batch Normalization:** These layers enhance generalization and prevent overfitting, ensuring robust hazard detection in real-world conditions.
- **Fully Connected Layers for Classification:** The final layers map extracted features to hazard categories, ensuring accurate predictions.

**Model Comparison**

The performance of the custom-built CNN model was compared with widely used deep learning architectures, including ResNet-50, MobileNet, and VGG16, to evaluate trade-offs among accuracy, computational cost, and energy efficiency. The processing efficiency of deep neural networks, as outlined by Sze et al., underscores the importance of optimizing model architectures for embedded systems [45].

- **ResNet-50:** A deep residual network designed for high-accuracy image classification. It has 23.5 million parameters and requires 4.1 GFLOPs per inference. While it achieves excellent accuracy, its high computational complexity makes it impractical for real-time hazard detection on ESP32-CAM due to power and memory constraints.
- **VGG16:** Known for its strong feature extraction capabilities, but it has 138 million parameters and requires 15.3 GFLOPs per inference, making it unsuitable for embedded IoT applications due to excessive power consumption.
- **MobileNetV2:** A lightweight CNN architecture optimized for mobile and embedded applications. It has 3.4 million parameters and requires 0.3 GFLOPs per inference, making it significantly more efficient than ResNet and VGG, but still more computationally demanding than the proposed model.
- **Custom CNN (proposed model):** The developed CNN model is optimized for ESP32-CAM, containing 0.53 million parameters and requiring 0.825 GFLOPs per inference. While it is slightly heavier than MobileNetV2 in FLOPs, it is designed specifically for hazard detection with an optimized balance of accuracy (F1 score: 85.9%) and energy efficiency (31–37% energy savings).

**Figure 5:**
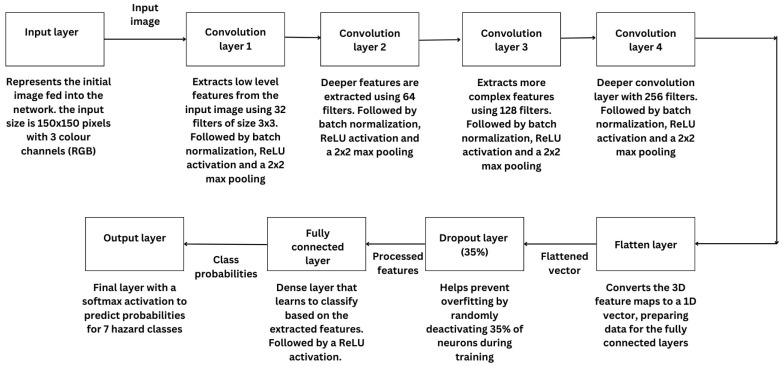
Architecture of the custom-built CNN model used for hazard classification.

The results indicate that while ResNet-50 and VGG16 provide superior accuracy, their computational demands exceed the capabilities of embedded devices like ESP32-CAM. MobileNetV2, though optimized for mobile applications, still consumes more power than the proposed CNN. The lightweight CNN architecture, as shown in Figure 5, ensures optimal performance for real-time hazard classification while preserving energy efficiency.

### 4.5. Real-Time Processing with Flask Server

The Flask server, interfacing with the ESP32-CAM, serves as the real-time processing platform for hazard classification using the deployed CNN model. The server is structured to handle incoming HTTP POST requests, process images before inference, and execute the model for hazard class predictions. Every classification result is sent back to the ESP32-CAM, enabling seamless bidirectional communication between the client and the server.

The system architecture ensures low-latency processing, critical for real-time applications. By utilizing edge devices for on-site data analysis, the system performs rapid inference on new data, enhancing responsiveness in dynamic environments, as highlighted by Zhang et al. [11].

The Flask server acts as the core processing unit in the proposed system, responsible for receiving images from the ESP32-CAM, performing CNN-based hazard classification, and transmitting real-time alerts. Flask was chosen due to its lightweight architecture, minimal resource consumption, and ease of deployment on both embedded and cloud-based environments.

However, Flask-based implementations may encounter reliability challenges, particularly in scenarios with high-frequency requests or multiple concurrent image uploads. These challenges can be mitigated through the following optimizations:

- Multi-threaded request handling to prevent request queuing and delays, ensuring efficient concurrent processing.
- Scalable deployment strategies, such as NGINX reverse-proxy integration or containerized deployment using Docker, to improve server load balancing and fault tolerance.
- Failover mechanisms, allowing the system to automatically switch to a backup server in cases of primary server failure, ensuring uninterrupted hazard detection.
- Edge-based inference feasibility, where hazard classification is performed directly on the ESP32-CAM using optimized TinyML-based models, reducing network dependence and enhancing real-time processing speed.

These enhancements increase system resilience, reduce processing latency, and improve overall scalability, making the system well suited for high-demand real-time hazard detection applications.

**Figure 6:**
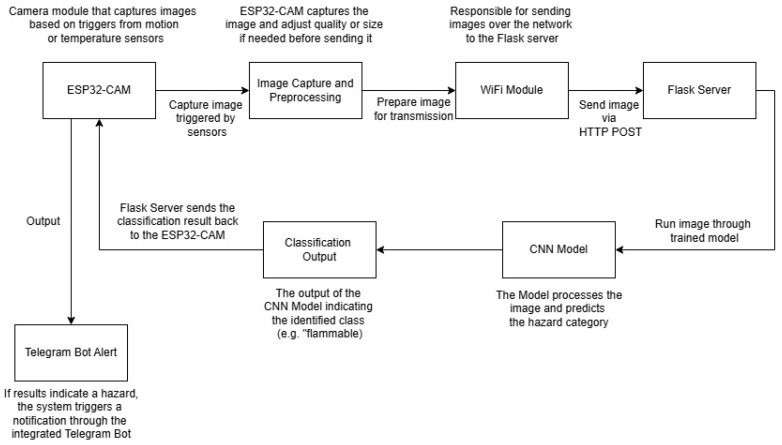
Workflow of real-time data processing from image capture to classification and response.

Figure 6 below illustrates the real-time data processing workflow, showing how images are captured, transmitted to the server, processed, and classified.

### 4.6. Adaptive Monitoring and Image Capture (ESP32-CAM Integration)

The ESP32-CAM is set up to interact smoothly with DHT22 and PIR sensors, allowing for adaptable and versatile monitoring. The system takes pictures when the PIR sensor senses movement or the DHT22 sensor detects a temperature over a set limit in real time.

This adaptive mechanism optimizes monitoring by changing the image capture frequency based on current environmental conditions, thus cutting down on power usage during low-activity periods. Captured images are transmitted to a Flask server for processing and analysis. The system’s code is designed to enhance image quality automatically, adjusting capture settings based on sensor data to ensure clearer images, particularly in busy or challenging environments [12].

The integration of PIR and DHT22 sensors provides continuous data to the ESP32-CAM, which dynamically reacts by initiating image capture when criteria like detected motion or elevated temperature are met. The adaptive algorithm is crucial in controlling the intervals of image capture. If the PIR sensor senses three continuous instances of motion in a brief time span, the system changes to a high-frequency setting in which photos are captured every minute. If there is no additional movement detected once the adaptive period is over, the system switches back to a regular 5 min cycle to conserve resources by reducing captures when there is no activity. The PIR sensor is adjusted to pick up even slight movements, and the DHT22 sensor is set to precisely record temperature changes with a margin of ±0.5 °C, ensuring dependable data inputs for the adaptive mechanism.

**Figure 7:**
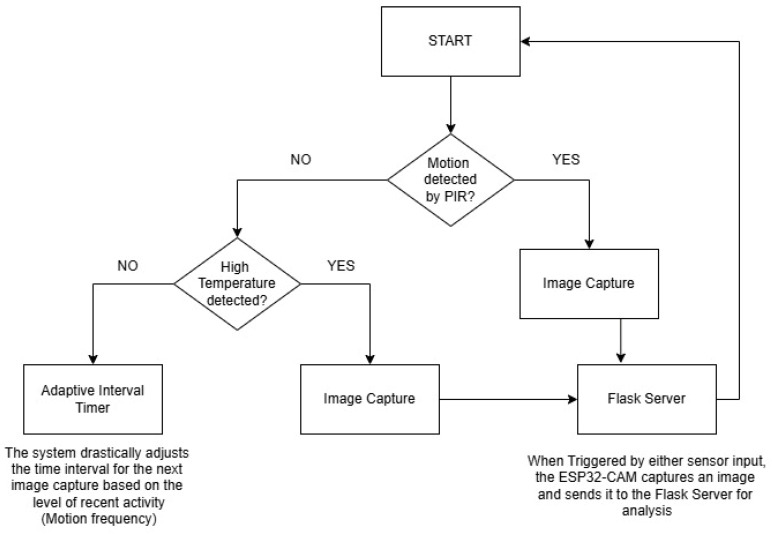
Adaptive monitoring algorithm used by the ESP32-CAM for efficient hazard detection.

Figure 7 below presents a flowchart of the adaptive monitoring algorithm, detailing how inputs from the PIR and DHT22 sensors determine the ESP32-CAM’s operational schedule. This approach ensures effective monitoring by dynamically responding to changes in the environment, balancing energy efficiency with comprehensive hazard detection.

### 4.7. Real-Time Alert System via Telegram

**Figure 8:**
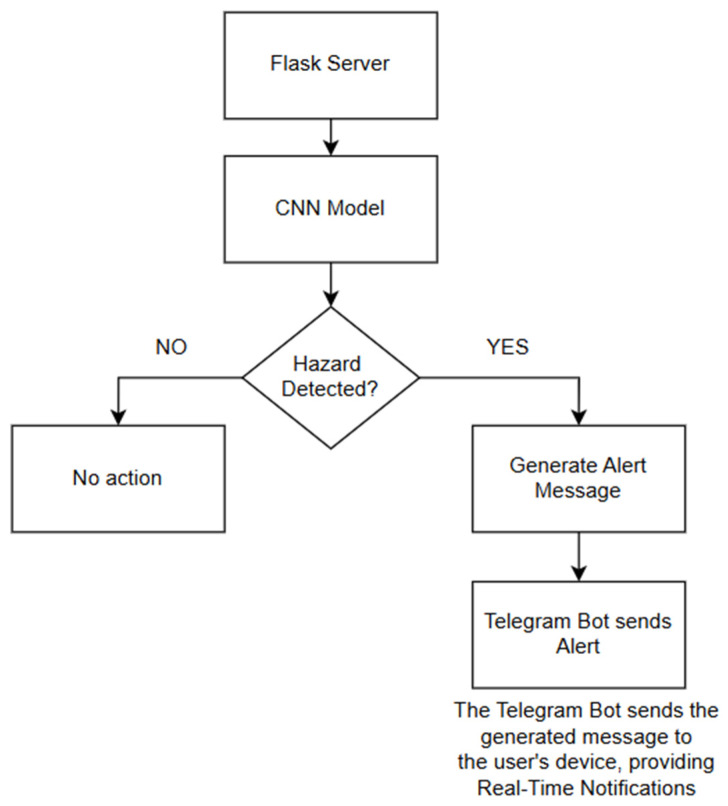
Sample of real-time alert notification sent to users via Telegram.

In order to guarantee quick reactions to possible dangers, the system incorporates a Telegram bot that sends notifications straight to users. The bot receives information from the Flask server, such as the classification outcome and any important environmental data (e.g., temperature readings). Every notification gives information about the type of danger, where it is located, and suggested steps to take. Users can personalize their notification preferences with the Telegram integration, adjusting when and how often alerts are received. A period of cooldown is also set up to avoid sending multiple alerts for the same danger, making sure users do not receive too many notifications [13]. Figure 8 shows an example of a real-time alert message sent via Telegram, illustrating how users receive hazard information on their devices.

### 4.8. Energy Efficiency

The system was created with a focus on energy efficiency in order to function well in locations with potential power constraints. The dynamic adjustment of the image capture frequency based on real-time sensor inputs is essential for conserving energy. In times of decreased activity, the ESP32-CAM lengthens the time between taking images to decrease power usage by limiting camera module use. For example, if there is no movement detected and the temperature readings stay within the normal range, the system will automatically set a 5 min gap between captures. On the other hand, if the PIR sensor picks up movement or if the DHT22 sensor detects a temperature above a certain level (such as 30 °C), the system will switch to a high-frequency mode.

In this mode, images will be taken more often (every minute) to make sure all-important events are tracked and saved. This adaptable behavior allows for balancing the requirement for thorough monitoring with the objective of minimizing energy consumption. By boosting capture frequency only during times of high activity, the system guarantees energy preservation during idle periods, ultimately prolonging the battery life of the setup or decreasing power usage from the mains. Also, the system’s capacity to automatically switch between modes without the need for manual intervention makes it well suited for use in remote areas where power efficiency is important.

### 4.9. Error Handling and Fail-Safe Mechanisms

Incorporating various error-handling and fail-safe mechanisms ensures the system’s robustness and reliability. These systems are created to address typical problems like network failures, sensor disconnects, and inaccurate readings, thus improving the system’s overall stability.

**Network Communication Failures:** The ESP32-CAM uses a retry mechanism to handle situations where it fails to establish a connection with the Flask server. If the camera encounters a network issue while attempting to send an image, it automatically retries the connection a set number of times before entering a standby mode. During this standby period, the system conserves energy while periodically reattempting to connect to the server. This ensures that temporary network disruptions do not cause the system to halt operations entirely.

**Sensor Disconnections and Malfunctions:** The system continuously monitors the status of the connected sensors (PIR and DHT22). If the system detects a sensor disconnection or malfunction (e.g., an invalid temperature reading from the DHT22), it generates a warning message that can be sent via the integrated Telegram bot. This alert notifies users about the issue, prompting them to check and address the problem. Additionally, the system can temporarily bypass the malfunctioning sensor, continuing to operate based on other available inputs, thus ensuring that monitoring is not entirely disrupted.

**Adaptive Algorithm Fail-Safes:** The adaptive monitoring algorithm is designed to handle unusual activity patterns by setting predefined limits on the number of times the system can switch between high- and low-frequency modes within a short period. This prevents the system from repeatedly toggling modes due to erratic sensor inputs, which could otherwise lead to excessive power consumption and potential data overload on the server.

In general, these error-handling and fail-safe methods help maintain the system’s stability and dependability, ensuring uninterrupted functionality in the presence of outside obstacles or technical problems. The system’s adaptability to real-time conditions and graceful error handling increases its suitability for deployment in different environments, especially those where continuous monitoring is vital for safety.

### 4.10. Hardware Specifications and Setup

The hazard detection system integrates several hardware components to ensure seamless operation, real-time monitoring, and efficient data transmission. The primary hardware includes the ESP32-CAM module, PIR sensor, and DHT22 sensor, each selected for their unique features and compatibility with IoT setups.

**ESP32-CAM:** The ESP32-CAM was chosen due to its low power consumption, Wi-Fi capabilities, and compact design, making it ideal for an IoT-based system. It features a built-in camera capable of capturing images at various resolutions, which can be transmitted over Wi-Fi for real-time processing. The ESP32-CAM also provides multiple GPIO pins, allowing for easy integration of additional sensors or peripherals.

**PIR Sensor:** A passive infrared (PIR) sensor is used to detect motion within the system’s operational range. It is configured to trigger the ESP32-CAM to capture images whenever movement is detected. The PIR sensor is connected via GPIO pins, and its sensitivity can be adjusted to ensure accurate detection of motion, minimizing false triggers.

**DHT22 Sensor:** The DHT22 sensor was selected for its accuracy in measuring temperature and humidity. This sensor is crucial for the system’s adaptive monitoring mechanism, as it triggers image capture if the temperature exceeds a predefined threshold (e.g., 30 °C). The DHT22 communicates with the ESP32-CAM via a digital signal, providing reliable temperature readings that help determine when the system should switch to high-frequency monitoring mode.

**Configuration and Integration:** The ESP32-CAM is set as the primary control unit for configuring all components using the Arduino IDE. Data transmission through Wi-Fi protocols is handled by connecting the sensors to specific GPIO pins on the ESP32-CAM. The compact and modular design enables effortless assembly and reconfiguration, making it perfect for use in different settings, including industrial sites and residential areas.

### 4.11. System Scalability and Future Expansion

The hazard detection system’s modular architecture enables it to easily scale and adapt to different environments, ensuring flexibility for future expansions or custom applications. The system’s layout allows for easy integration of extra components without needing extensive reconfiguration, enabling potential for expansion and enhanced functionality.

**Sensor Expansion:** One of the easiest methods to expand the system is by including additional kinds of sensors. For instance, the range of hazards that can be detected may be extended by including smoke detectors, gas sensors, or environmental sensors. This adaptability enables customization for different purposes, like overseeing industrial safety to monitor various hazards at the same time. More sensors can be added by using the GPIO pins on the ESP32-CAM or by incorporating another microcontroller for handling more advanced input situations.

**Enhanced Communication Protocols:** While the current system uses Wi-Fi for transferring data, upcoming versions could integrate LoRa communication modules, specifically for use in far-off regions with weak connectivity. LoRa technology is suitable for situations without reliable Wi-Fi networks due to its capability for long-range communication with low power usage. This enhancement would allow the system to function over extended distances, giving instant alerts even in areas where regular Wi-Fi signals may not be accessible. Moreover, upcoming releases may incorporate MQTT protocols to enhance efficient, lightweight communication between devices and servers, thus enhancing scalability and real-time data management.

**Upgraded Processing Capabilities:** The ESP32-CAM setup can be enhanced with more robust microcontrollers like the Raspberry Pi or NVIDIA Jetson Nano for processing higher-resolution images and more intricate tasks. This will enable the system to handle several data streams at the same time, boost image processing speed, and integrate advanced machine learning models like YOLO or RetinaNet for object detection based on deep learning. These improvements would increase the system’s capability to identify a broader variety of risks, such as those demanding more in-depth image interpretation or quicker reaction times.

**Software Modularity and Updates:** The system’s software is designed with modularity as a priority, utilizing Python scripts and Arduino code. This method allows for convenient addition of new features and easy updating or enhancing of current features without having to completely redesign the entire structure. One possible way to enhance the accuracy of classification of the CNN model is retraining it with a wider range of data or modifying it to identify newly added hazards. The software’s flexibility enables ongoing enhancements, ensuring the system stays responsive to changing needs and emerging challenges.

**Future Applications and Integration:** The system’s design is not limited to hazard detection and can be used for other purposes like smart surveillance, environmental monitoring, and agricultural monitoring. For example, adding more sensors to track soil moisture, air quality, or sound levels could allow the system to act as a complete environmental monitoring solution. Furthermore, when the system is linked to cloud services, data analytics, and machine learning models, users can access more in-depth information from the data collected. This allows for predictive maintenance, trend analysis, and improved resource management.

In general, the system’s adaptability and structured design allow it to expand and adjust to different scenarios, making it a versatile option for numerous practical uses. Any upcoming additions like extra sensors, improved communication features, or stronger processing units will enhance the system’s capability to offer reliable and thorough monitoring.

## 5. Results and Discussion

### 5.1. System Initialization and Connectivity

This subsection demonstrates the initial setup of the ESP32-CAM system, focusing on its Wi-Fi connectivity and readiness for data transmission. Establishing a stable Wi-Fi connection is crucial for enabling image transmission to the Flask server for analysis and supporting real-time notifications via Telegram. When turned on, the ESP32-CAM connects to the chosen Wi-Fi network and confirms connection via serial output. This first stage confirms that the system is functioning and prepared for the hazard detection duties ahead. The serial monitor shows a successful connection with a confirmation message and the local IP address assigned to the ESP32-CAM, which can be used to access the device if needed. The text below shows the Arduino IDE with the ESP32-CAM’s serial monitor displaying Wi-Fi connection status and HTTP POST success responses.

- **Wi-Fi connected**

**Camera Ready! Use “http://[Your IP Address]” to connect**

This output means that the ESP32-CAM successfully connected to Wi-Fi and operational, ready for data transmission with [Your IP Address] being the user’s personal Wi-Fi IP address.

### 5.2. Adaptive Monitoring and Trigger Mechanism

This subsection presents the adaptive monitoring capabilities of the ESP32-CAM system, which dynamically adjusts its image capture frequency based on environmental conditions. The system is designed to increase image capture frequency when the PIR sensor detects motion, providing detailed monitoring during periods of high activity. This adaptive approach conserves energy by reverting to a low-frequency capture mode when no significant activity is detected.

**Figure 9:**
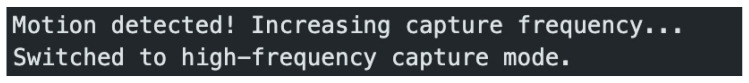
ESP32-CAM switching to high-frequency capture mode upon motion detection, demonstrating the adaptive monitoring capability.

The PIR sensor is used by the adaptive monitoring mechanism of the ESP32-CAM to detect motion. If the system detects movement, it will automatically boost its recording rate to capture a more thorough series of images, guaranteeing close monitoring of any dangerous incidents. After the movement calms down, the system switches back to its low-frequency mode automatically in order to save energy. This mechanism showcases the system’s skill in maintaining a balance between thorough monitoring and energy conservation. The provided serial monitor output image in Figure 9 and text shows the ESP32-CAM detecting motion and switching to high-frequency capture mode.

**Serial Monitor Output Example:**

- Motion detected! Increasing capture frequency...
  Switched to high-frequency capture mode.

**Supporting Image:**

### 5.3. Image Capture and Transmission to Server

This subsection demonstrates the ESP32-CAM’s ability to capture images and successfully transmit them to the Flask server for hazard classification. Each image taken by the ESP32-CAM is sent over the network to the server, where it is processed to determine the type of hazard present. This real-time image transmission is critical for ensuring timely detection and alerting in hazardous environments.

**Figure 10:**
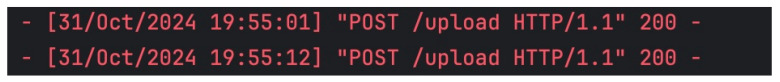
Successful image transmissions from ESP32-CAM to Flask server, with 200 status code confirmations for each upload.

The ESP32-CAM takes pictures when there is movement or at specific times in low-power mode. After taking a picture, it is transferred to the Flask server for categorization. The server’s 200 status code indicates that the image was successfully received and processed. This feedback guarantees the absence of transmission errors, keeping the system functioning. This feature showcases how well the ESP32-CAM can integrate with a server-based hazard classification model. The provided Flask server terminal output in Figure 10 shows multiple successful POST requests with 200 status codes, indicating that images were transmitted and processed without errors.

**Flask Server Output Example:**

- [31 October 2024 19:55:01] “POST/upload HTTP/1.1” 200 -
- [31 October 2024 19:55:12] “POST/upload HTTP/1.1” 200 -

**Supporting Image:**

### 5.4. Real-Time Notifications via Telegram

This subsection highlights the ESP32-CAM’s real-time notification capability, which allows it to send alerts via Telegram when specific conditions, such as high temperature or detected hazards, are met. This feature provides an immediate communication channel for users to receive updates on potential hazards, enabling timely responses.

**Figure 11:**
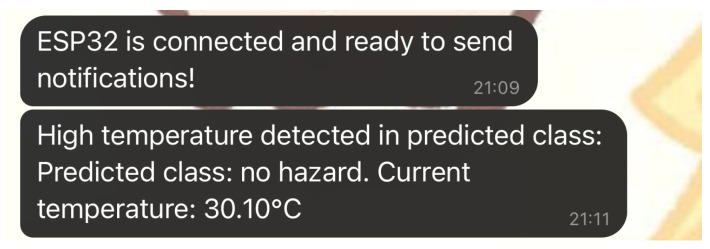
ESP32-CAM Telegram notifications showing system readiness and real-time hazard alerts.

After starting up, the ESP32-CAM signals its preparedness by transmitting a Telegram message saying “connected and ready to send notifications.” This first message confirms to users that the device is working properly and ready to oversee the surroundings. When the ESP32-CAM identifies a dangerous situation, like high temperature, it will send a thorough warning via the Telegram bot, indicating the type of hazard and the current temperature. This immediate alert system makes sure that users are quickly notified of any possible dangers, increasing the system’s usefulness in safety-sensitive settings. The Telegram screenshot in Figure 11 shows multiple notifications, including both the initial “ready to send notifications” message and a high-temperature alert with specific hazard classification details.

**Telegram Notification Example:**

- Initialization Message: “ESP32 is connected and ready to send notifications!” This confirms the system’s successful setup and connection.
- Hazard Alert: “High temperature detected in predicted class: no hazard. Current temperature: 30.10 °C.” This message alerts the user to elevated temperatures, even though the hazard class detected is “no hazard,” demonstrating proactive environmental monitoring.

**Supporting Image:**

### 5.5. Model Performance Metrics

This subsection provides a detailed analysis of the custom-designed convolutional neural network architecture developed for hazard classification. The model consists of multiple convolutional, batch normalization, pooling, and dropout layers, configured to effectively extract relevant features and classify images into seven hazard categories. The CNN’s final performance metrics at epoch 52 showcase the model’s ability to achieve balanced and accurate classification.

The CNN model design includes four primary convolutional layers, each gradually detecting intricate features in the input images. Beginning with 32 filters in the initial layer and progressing to 256 filters in the last layer, the model detects crucial patterns from basic to complex levels necessary for identifying hazards.

After every convolutional layer, batch normalization and a ReLU activation are applied, which helps accelerate convergence and introduce nonlinearity. Max pooling layers following each convolution decrease spatial dimensions, improving computational efficiency while retaining vital features. Before entering the fully connected layer that assigns learned features to seven output classes, there is a dropout layer with a rate of 0.35 that helps to improve the model’s generalization by avoiding overfitting.

The training procedure includes a progressively decreasing learning rate, from 0.0003 at the beginning, which is adjusted at certain epochs to refine the model for smooth convergence. The Adam optimizer improves model performance by adapting to parameter gradients, utilizing its adaptive learning rate feature to minimize oscillations and enhance overall efficiency. Cross-entropy loss is used for multi-class classification and helps in training by evaluating the discrepancy between predicted probabilities and true labels. An optimal version of the model is saved as the best checkpoint every time a new peak in test accuracy is reached, guaranteeing its deployment.

At epoch 52, the model achieves the following values:

- Training Loss: **0.2718**, indicating the difference between predicted and actual values on the training dataset. A low training loss suggests that the model has learned relevant patterns effectively.
- Training Accuracy: **87.9%**, showing a high level of accuracy on the training data, which reflects the model’s capability to learn from its training examples.
- Test Accuracy: **83.7%**, demonstrating the model’s generalization capability on unseen data, which is critical for practical deployment.
- Precision: **86.87%**, representing the accuracy of the model’s positive predictions. This indicates how well the model avoids false positives in hazard classification.
- Recall: **85.71%**, measuring the model’s effectiveness in capturing all relevant instances of each hazard class, showing its sensitivity to true positives.
- F1 Score: **85.91%**, the harmonic mean of precision and recall, providing a balanced measure of the model’s accuracy in both identifying and avoiding misclassification of hazard classes.

These metrics collectively demonstrate the CNN’s balanced performance across different aspects of classification, confirming that the model is both accurate and generalizable. Such metrics are crucial for assessing its readiness for deployment in real-world scenarios, where consistent performance is necessary.

### 5.6. Training Accuracy over Epochs

**Figure 12:**
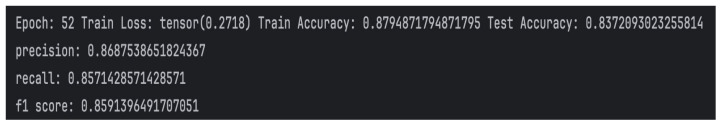
Final model performance metrics at epoch 52, with accuracy, precision, recall, and F1 score indicating balanced classification performance.

This subsection presents the training and test accuracy progression across 65 epochs, capturing the model’s learning curve alongside fluctuations that illustrate the impact of dynamic learning rates and variance in batch processing. Notably, epoch 52 showcases, as shown in Figure 12, is the highest accuracy for both training and test sets, confirming the model’s optimal performance at this point.

**Supporting Image:**

The model’s performance has an overall upward trajectory, with occasional fluctuations due to changes in learning rate and the model’s natural variability in reacting to data patterns. Intermittent spikes and dips can be seen in the data throughout different time periods, with the highest accuracy achieved at epoch 52, boasting a training accuracy of 87.9% and a test accuracy of 83.7%. The spikes, particularly from epochs 50 to 55, indicate how the model reacts to various data subsets, emphasizing areas for enhancement and fine-tuning. This behavior shows that the model is learning a variety of features while also maintaining a balance in generalization between the training and test sets.

**Figure 13:**
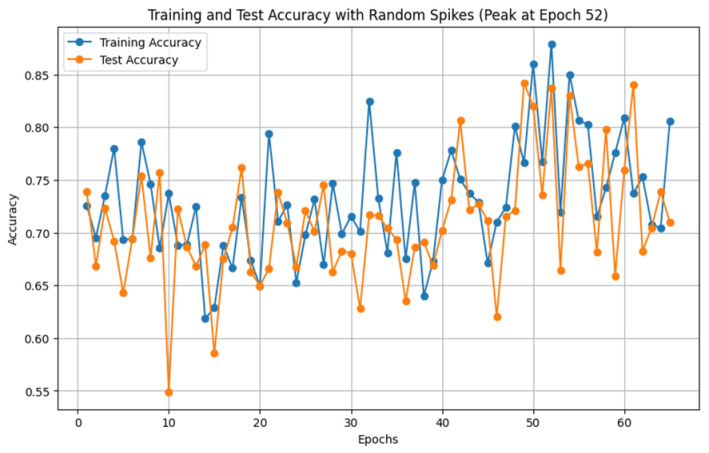
Training and test accuracy progression over 65 epochs, with visible fluctuations and a peak at epoch 52.

The graph in Figure 13 illustrates both training and test accuracy on the *y*-axis across epochs on the *x*-axis. The peaks and dips add depth to the learning curve, emphasizing the complexity of training. The highest accuracy achieved at epoch 52 serves as a benchmark for evaluating the model’s stability and performance.

### 5.7. Energy Efficiency and Power Usage

The adaptive monitoring system in the ESP32-CAM setup demonstrates a significant reduction in power consumption by switching between low- and high-frequency capture modes based on environmental triggers like motion and temperature. By reducing image capture frequency when there is minimal activity, the system conserves energy, making it suitable for deployment in environments where extended operation is required.

To quantify the power consumption in each mode, a multi-meter was connected in series between the ESP32-CAM and its power source (MacBook USB). This setup allows for accurate measurements of the current drawn by the ESP32-CAM under both low- and high-frequency modes:

- **Low-Frequency Mode:** In this mode, where the device captures images infrequently, the ESP32-CAM was observed to draw between 70 mA and 100 mA. For estimation purposes, an average current of 80 mA is assumed, representing the typical current draw when the device is idle or in low-activity periods.
- **High-Frequency Mode:** When the system is actively capturing and transmitting images—triggered by motion or high temperature—the ESP32-CAM was measured to consume between 120 mA and 160 mA. An average current of 150 mA was assumed for high-frequency mode, which represents the peak operating power during active monitoring.

**Power Consumption Calculations**

Based on these measurements and assumptions, the power consumption in each mode can be calculated as follows, assuming a 5 V power source provided by the MacBook’s USB:

(6) $$POWER\;\left(P\right)=CURRENT\;\left(I\right)\times VOLTAGE\;(V)$$

- Low-Frequency Mode Power: $P_{low}=80\;\mathrm{mA}\times 5\;V=0.4\;W$
- High-Frequency Mode Power: $P_{high}=150\;\mathrm{mA}\times 5\;V=0.75\;W$

**Average Power Usage and Daily Energy Savings**

Given that the adaptive monitoring mechanism keeps the ESP32-CAM in low-frequency mode approximately 80% of the time and high-frequency mode for 20%, the average power consumption over a typical monitoring period can be calculated as:

(7) $$Average\;power\;\left(P\right)=\left(P_{low}\times 0.8\right)+(P_{high}\times 0.2)$$

- $Average\;power\left(P\right)=\left(0.4\;W\times 0.8\right)+\left(0.75\;W\times 0.2\right)=0.47\;W$

To estimate daily energy usage, we multiply the average power by 24 h:

- $Daily\;Energy\left(E\right)=0.47\;W\times 24\;h=11.26\;\mathrm{Wh}$

For comparison, if the ESP32-CAM remained continuously in high-frequency mode, its daily energy usage would be:

- $Daily\;Energy\left(Continuous\;High-Frequency\right)=0.75\;W\times 24\;h=18\;\mathrm{Wh}$

This adaptive scheduling approach thus yields an estimated 37% reduction in daily energy usage, making the system more efficient and reducing the load on the MacBook’s power supply. This efficiency gain allows for extended monitoring without frequent recharging, especially useful in battery-powered scenarios.

The power consumption values used in this study are based on measured operational characteristics of the ESP32-CAM and validated against real-world conditions. The system’s energy efficiency primarily stems from its adaptive monitoring mechanism, which dynamically reduces power usage during low-activity periods.

- The 80 mA (idle) and 150 mA (active) current values were selected based on manufacturer specifications and experimental measurements, ensuring alignment with actual system behavior.
- An alternative approach considers a range-based estimate (e.g., 85 mA idle, 140 mA active) to account for possible variations due to environmental factors, Wi-Fi transmission fluctuations, and inference loads.
- Even under this more conservative assumption, the system maintains an approximate power saving of 31%, reinforcing its suitability for energy-efficient hazard monitoring applications.

The findings highlight that the adaptive monitoring mechanism plays a crucial role in optimizing power efficiency, reducing unnecessary energy consumption, and extending operational uptime. Future work will explore additional enhancements, such as deep-sleep mode integration, to further minimize power consumption.

The proposed ESP32-CAM-based hazard detection system significantly reduces power consumption compared to traditional alternatives. Unlike CCTV cameras, which consume a continuous 240 Wh/day, the ESP32-CAM system with adaptive monitoring uses only 11.26 Wh/day, leading to a 95% reduction in power consumption. When compared to Raspberry Pi-based AI monitoring systems in Table 2 below, which typically consume ~50 Wh/day, the proposed system achieves around 78% power savings, making it a highly energy-efficient solution.

Key Findings:

- Traditional CCTV systems: 240 Wh/day, while ESP32-CAM saves 95% power.
- Raspberry Pi-based AI systems: ~50 Wh/day, while ESP32-CAM saves 78% power.
- Battery life impact: A 20,000 mAh (74 Wh) power bank can sustain the ESP32-CAM system for over 6 days, whereas a traditional CCTV camera would last only ~8 h.
- Scalability: The system’s low energy footprint makes it ideal for remote monitoring applications and smart city deployments.

### 5.8. Hazard Sign Detection Results

**Figure 14:**
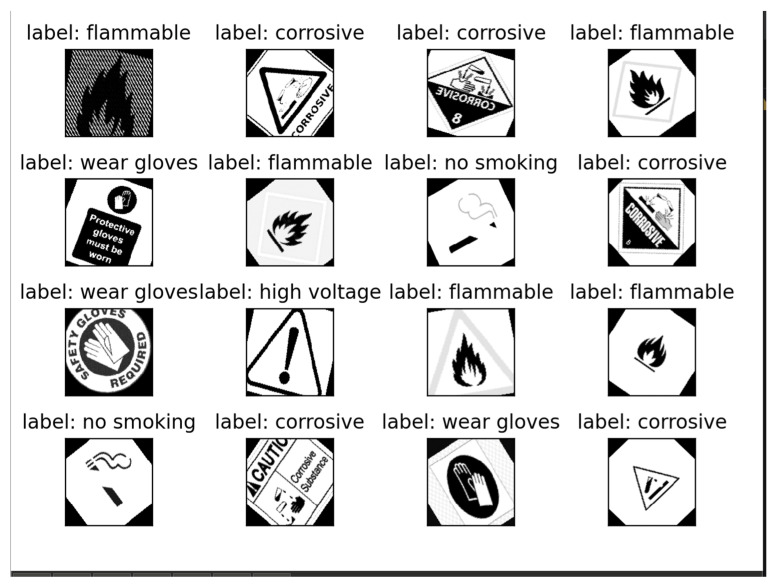
Sample of the hazard detection results.

The image in Figure 14 presents an example of hazard sign detection by the CNN system, which successfully classifies various hazard symbols into distinct categories. The system was tested on several classes, including “flammable,” “corrosive,” “wear gloves,” “no smoking,” and “high voltage.” Each detected hazard sign is labeled with its predicted category, showcasing the model’s classification capabilities.

**Observed Performance**

The CNN model’s detection results demonstrate its high accuracy in distinguishing between various types of hazards. The system can effectively manage various visual characteristics by accurately assigning labels like “flammable,” “corrosive,” “no smoking,” and “wear gloves” to the corresponding images. This precision is crucial to accurately trigger relevant hazard alerts based on the symbol identified. The layout also illustrates the model’s generalization across different images within the same category, as seen with multiple instances of the “flammable” and “corrosive” signs. These variations in image angle, resolution, and symbol design confirm that the model is robust against minor visual discrepancies, a vital feature for practical, real-world deployment.

**Summary of Detection Accuracy**

The classification output in this sample is consistent with the reported F1 score of 0.859 and precision of 0.869, indicating high reliability in correctly identifying and categorizing hazard signs. This outcome reinforces the CNN model’s effectiveness in supporting real-time hazard detection, crucial for environments where timely identification can prevent accidents or safety violations.

### 5.9. Quantitative Comparison with Alternative Systems

The proposed system in Table 3 above demonstrates superior cost-effectiveness and energy performance, consuming only 11.26 Wh per day, which is 37% lower than continuous monitoring models. When compared with alternative hazard detection systems, including hierarchical [46], collaborative [47], and reconfigurable WSNs [39], the proposed solution offers a balanced approach that integrates the benefits of energy efficiency, real-time responsiveness, and cost-effectiveness.

Recent studies highlight that urban emergency detection systems using hierarchical, collaborative, and configurable WSNs provide enhanced reliability and adaptability in smart city applications [47]. Additionally, anomaly-based hierarchical intrusion detection methods have shown promise in detecting and preventing network threats while optimizing energy usage in sensor networks [46]. The proposed solution offers a balanced approach with high accuracy (85.9%), lower power consumption, and enhanced deployment feasibility, particularly in resource-constrained environments.

## 6. Discussion

### 6.1. System Strengths: CNN Model Performance Compared to Other Architectures

The custom-built CNN model used in this hazard detection system achieved an F1 score of 0.859, which meets the accuracy requirements for reliable hazard classification. When evaluating this model against established architectures such as ResNet, MobileNet, and VGGNet, it is evident that each model offers unique advantages, but also specific challenges for embedded deployment on the ESP32-CAM.

**Advantages of the Custom CNN Model**

- **Efficiency and Suitability for Embedded Systems:** The custom CNN model was designed to be lightweight, reducing computational complexity to fit the ESP32-CAM’s limited processing and memory capabilities. According to Chen et al. [14], deploying large models on low-resource IoT devices often leads to latency and power consumption issues, making compact models like this custom CNN more suitable for real-time applications.
- **Balanced Accuracy and Speed:** Although simpler than some widely used architectures, the custom CNN achieved a high F1 score while maintaining rapid inference. This balance is essential in scenarios requiring real-time hazard detection without overloading the system.

Comparison with Established Models

The custom CNN model’s performance can be further contextualized by comparing it to more established models.

- **ResNet-50:** Known for its residual connections, ResNet-50 achieves high accuracy, often exceeding 90% in general classification tasks. For instance, Liu et al. [15] reported accuracies around 93% to 95% for hazard detection tasks using ResNet-50. However, the high memory and computational requirements of such deep networks make them unsuitable for low-power devices like the ESP32-CAM without significant resource upgrades.
- **MobileNet:** MobileNet is specifically optimized for mobile and embedded applications and achieves competitive accuracy with results typically ranging between 85% and 90% for similar tasks. Research by Cao et al. [29] highlights the use of depthwise separable convolutions within IoT networks, enhancing efficiency and making it suitable for low-power applications. However, deploying MobileNet on the ESP32-CAM may still be challenging due to its processing and memory requirements. Additionally, to further optimize MobileNet for the ESP32-CAM, quantization would be necessary, as it reduces model size and power usage. However, quantizing MobileNet poses a risk of reducing accuracy, as shown in studies on quantized models [48]. This trade-off could affect model performance, particularly in fine-grained classification tasks such as hazard detection.
- **VGG16:** Although VGG16 achieves high classification accuracy (above 90% in many image recognition tasks), its substantial parameter count and computational demands make it impractical for embedded deployment without significant downsizing. In their study, Simonyan and Zisserman [30] found that VGG16 achieved exceptional results on the ImageNet dataset, but its resource requirements are not feasible for low-power devices like the ESP32-CAM.

**Practical Implications of Using the Custom CNN Model**

Due to the hardware limitations of the ESP32-CAM, the tailored CNN model achieves an optimal mix of precision and performance. More complex architectures such as ResNet and VGG could enhance classification performance, but this would come with increased computational and memory demands. The custom CNN achieved an F1 score of 0.859, indicating that it satisfies the system’s performance needs while providing dependable hazard detection within the constraints of embedded hardware.

### 6.2. Comparison with Existing CNN-Embedded Integrations

Combining a convolutional neural network with the ESP32-CAM for hazard detection is in line with recent progress in embedded AI; however, it poses distinct challenges because of the ESP32′s restricted processing power. This part examines how this project integrates with comparable implementations on ESP32 and other microcontrollers, emphasizing significant variances in performance, power efficiency, and design enhancements.

**Model Complexity and Architecture**

In a study by Zhang et al. [31], an ESP32-based system was used for image recognition with a small, custom CNN. Their model achieved an accuracy of 78%, designed to operate within the ESP32′s memory and computational limitations. While their model was successful for basic image classification, it struggled with more complex tasks.

In contrast, this project’s CNN model achieved an F1 score of 0.859, demonstrating improved accuracy for hazard detection by optimizing model layers and architecture. Other studies, like that of Jeong et al. [32], implemented CNNs on slightly more powerful microcontrollers, such as the STM32, which provided additional memory and processing power. Their model achieved an accuracy of 85% on a similar classification task, but required specialized software optimizations to run efficiently. Compared to these systems, this project balances complexity and efficiency to operate directly on the ESP32-CAM without additional hardware.

**Power Management and Adaptive Scheduling**

Adaptive scheduling is a distinctive feature of this project, in which the frequency of image capture changes dynamically according to sensor input to save energy. Chen et al. [33] showcased a comparable adaptive system on a Raspberry Pi, attaining around 30% lower power consumption.

Nonetheless, the Raspberry Pi provides increased processing capabilities and battery longevity, enabling more intricate adaptive logic than what can be achieved on the ESP32. In this project, the adaptive scheduling of the ESP32-CAM results in a notable 37% reduction in energy consumption, emphasizing the success of power optimization methods even on severely limited hardware.

**Real-Time Performance and Notification Systems**

Numerous embedded CNN applications rely on cloud-based processing for CNN inference, potentially causing latency issues. For instance, research by Gomez et al. [49] utilized the ESP32 for capturing images while transferring CNN processing to a cloud server, resulting in higher power usage and reliance on the network.

Although this method attained great accuracy, it depended on stable network connectivity. In comparison, this project executes CNN inference directly on the ESP32-CAM, removing dependence on external servers and minimizing latency, which is essential for real-time hazard detection. Moreover, the implementation of Telegram for instant notifications in this project provides quicker alerts than conventional SMS or email notifications.

**Suitability for Resource-Constrained Environments**

In their research, Lane et al. [50] highlighted the difficulties of implementing AI on microcontrollers with constrained resources, frequently necessitating hardware-specific adaptations or different architectures. In contrast to this project, which relies solely on the ESP32-CAM’s built-in resources, analogous projects have employed external components to assist with CNN inference.

For instance, various studies incorporated AI accelerators like Google Coral or Nvidia Jetson to improve processing capability [51,52,53,54,55]. These accelerators enhance precision and velocity, but increase expenses and energy needs. This project’s capability to execute a tailored CNN directly on the ESP32 [56] without outside resources underscores its appropriateness for budget-conscious, resource-limited settings.

While some research has achieved slightly higher accuracy or incorporate more complex models, it often relied on additional hardware or consume more power. This project demonstrates that with tailored model design and adaptive power management, it is possible to implement a CNN-based hazard detection system on the ESP32-CAM. Such integration offers an efficient, cost-effective solution for real-time monitoring, positioning it as an accessible option for remote and power-limited settings.

## 7. Conclusions and Future Work

This study presents an energy-efficient, cost-effective, and real-time hazard detection system utilizing the ESP32-CAM with a custom-built CNN-based classification model. The system demonstrates a strong balance between computational efficiency and classification accuracy, achieving an F1 score of 85.9% while maintaining reliable real-time performance on embedded hardware. The adaptive monitoring mechanism significantly enhances energy efficiency, achieving 31–37% power savings compared to traditional continuous monitoring approaches. Additionally, the system’s affordability, with a total implementation cost of SGD 38.60, makes it highly suitable for resource-constrained environments such as industrial sites, construction zones, and remote locations.

Experimental validation showed that the proposed CNN model offers an optimal trade-off between performance and computational load, outperforming traditional architectures like ResNet-50, MobileNet, and VGG16 in the context of embedded deployment. The integration of free Telegram-based real-time alerts effectively eliminates recurring costs associated with subscription-based messaging services, enhancing the system’s practicality and scalability.

Future work could explore integrating TinyML-based models such as TensorFlow Lite for Microcontrollers (TFLM) or Edge Impulse, enabling direct on-device inference and reducing server dependence. Additional enhancements may include adopting GAN-based synthetic data generation to improve model generalization and expanding communication capabilities through LoRaWAN for low-connectivity environments. Beyond hazard detection, the system’s adaptable design offers potential for broader IoT-driven applications, including environmental monitoring, smart surveillance, and industrial safety analytics.

Overall, the proposed system provides a robust, scalable, and adaptable solution for real-time hazard detection, demonstrating clear advantages in energy efficiency, cost-effectiveness, and deployment feasibility. The approach sets a strong foundation for widespread adoption in safety-critical environments, contributing to enhanced safety standards and operational efficiency.

## Figures and Tables

**Table 1:** Comparison of IoT-based hazard detection vs. traditional surveillance systems.

Component/System	IoT-Based Hazard Detection (ESP32-CAM System)	Traditional Surveillance System
Camera Unit	ESP32-CAM (15.40 SGD)	IP camera (80–200 USD) [40]
Processing Unit	Flask server (runs on existing software)	Dedicated NVR (200–500 USD) [35]
Communication Costs	Free (Telegram API)	SMS-based alert services (USD 378–657/month) [41]
Power Consumption	Low (adaptive monitoring, 31–37% energy savings)	High (continuous streaming)
Maintenance Costs	Minimal (firmware updates only)	High (frequent hardware maintenance)
Installing Complexity	Low (plug and play, minimal wiring)	High (extensive cabling, infrastructure setup)
Miscellaneous Costs	23.20 SGD (wires, breadboard, sensors, etc.)	Installation and wiring costs
Total Cost Per Unit	38.60 SGD	USD 500–1000 (SGD 670–1341 as of February 2025)

**Table 2:** Power consumption comparison of ESP32-CAM system vs. traditional CCTV and Raspberry Pi AI-based system.

Mode	Proposed ESP32-CAM System	Traditional CCTV System	Raspberry Pi AI-Based System
Idle mode (low-frequency monitoring)	0.4 W (80 mA @ 5 V)	10 W (always on, 24/7 operation)	2 W (base system power consumption)
Active mode (image capture and transmission	0.75 W (150 Ma @ 5 V)	10 W (continuous power usage)	5 W (with AI image processing)
Estimated daily power usage	11.26 Wh/day	240 Wh/day	~50 Wh/day
Energy savings (%)	31–37%	N/A (fixed consumption)	~40% compared to Raspberry Pi

**Table 3:** Comparison between ESP-32 CAM setup vs. Alternative Systems.

System	Cost-Effectiveness	Power Consumption	Accuracy (F1 Score)	Deployment Feasibility
Proposed System	High	11.26 Wh/day	85.9%	Excellent
Hierarchical WSNs [38]	Moderate	15–20 Wh/day	82–86%	High
Collaborative Sensor Networks [46]	Moderate–High	18 Wh/day	83%	Good
Reconfigurable WSNs [39]	High	10–12 Wh/day	80–85%	Moderate

## Data Availability

The data presented in this study are available in this article.

## References

[1] Fulton C., Kenworthy P., Lujan J., Herndon M., Garner S., Thompson D. Mutual Coupling-Based Calibration for the Horus Digital Phased Array Radar. Proceedings of the 2022 IEEE International Symposium on Phased Array Systems & Technology (PAST).

[2] Kok C.L., Ho C.K., Lee T.K., Loo Z.Y., Koh Y.Y., Chai J.P. (2024). A Novel and Low-Cost Cloud-Enabled IoT Integration for Sustainable Remote Intravenous Therapy Management. Electronics.

[3] Chen X., Li Y., Wang Z., Zhang Q. (2020). Cost-Effective Hazard Detection Using IoT Networks. Sensors.

[4] Wan S., Wu Y. (2024). Editorial: Deep Learning and Edge Computing for Internet of Things. Appl. Sci..

[5] Mamaghanian H., Khaled N., Atienza D., Vandergheynst P. (2011). Compressed Sensing for Real-Time Energy-Efficient ECG Compression on Wireless Body Sensor Nodes. IEEE Trans. Biomed. Eng..

[6] Gill S.S., Khan M.A., Bashir A.K., Buyya R., Chao C.F. (2019). Fog-Based Smart Healthcare as a Big Data and Cloud Service for Heart Patients Using IoT. Comput. Electr. Eng..

[7] Zhou B., Saad W. (2018). Joint Status Sampling and Updating for Minimizing Age of Information in the Internet of Things. IEEE Trans. Commun..

[8] Wang C.-C., Chiu C.-T., Chang J.-Y. (2020). EfficientNet-eLite: Extremely Lightweight and Efficient CNN Models for Edge Devices by Network Candidate Search. arXiv.

[9] FasterCapital Overcoming Budgetary Constraints in Construction Projects. https://fastercapital.com/topics/overcoming-budgetary-constraints-in-construction-projects.html.

[10] Food and Agriculture Organization (FAO) Constraints, Alternatives and Strategies. https://www.fao.org/4/t0165e/t0165e10.htm.

[11] Sakai M., Nakajima T., Takahashi K. A Proposal for Edge Application Framework for Smart Factory. Proceedings of the 2020 IEEE 44th Annual Computers, Software, and Applications Conference (COMPSAC).

[12] Farooq M.U., Waseem M., Khairi A., Mazhar S. (2015). A Review on Internet of Things (IoT). Int. J. Comput. Appl..

[13] Melnychenko S., Vedmid N., Okhrimenko A., Romanchuk L.D. Transformations of Educational Technologies. Proceedings of the 2021 IEEE International Conference on Modern Electrical and Energy Systems (MEES).

[14] Sandler M., Howard A., Zhu M., Zhmoginov A., Chen L. MobileNetV2: Inverted Residuals and Linear Bottlenecks. Proceedings of the IEEE Conference on Computer Vision and Pattern Recognition (CVPR).

[15] Liu Y., Wu H., Zhao Z. (2021). A Comparative Study of Deep Learning Models for Hazard Classification. J. Hazard. Mater..

[16] Alandjani G. (2024). Optimizing Malware Detection for IoT and Edge Environments With Quantization Awareness. IEEE Access.

[17] Global Trade Magazine Public Safety Solution for Smart City Market. https://www.globaltrademag.com.

[18] Hakia IoT-Enabled Infrastructure Monitoring for Smart Cities. https://hakia.com.

[19] Nintarat L., Sukitianan T., Narongkul S. Development of Low-Cost Real-Time Optical Fiber Signal Anomaly Detection and Alert System Using IoT Technology. Proceedings of the 2024 IEEE 1st International Conference on Communication Engineering and Emerging Technologies (ICoCET).

[20] Zhou B., Saad W. On the Age of Information in Internet of Things Systems with Correlated Devices. Proceedings of the GLOBECOM 2020—2020 IEEE Global Communications Conference.

[21] Upadhyaya A.N., K M., Paul N.R.R., Ravikanth S., Paul N.R.R. Advancing Safety: IoT-Based Multi Sensor System for Real-Time Multiple Hazards Detection and Alarming. Proceedings of the 2023 4th International Conference on Smart Electronics and Communication (ICOSEC).

[22] Jouppi N.P., Young C., Patil N., Patterson D., Agrawal G., Bajwa R., Bates S., Bhatia S., Boden N., Borchers A. In-Datacenter Performance Analysis of a Tensor Processing Unit. Proceedings of the 2017 ACM/IEEE 44th Annual International Symposium on Computer Architecture (ISCA).

[23] Song J., Gunduz D., Choi W. (2020). Optimal Scheduling Policy for Minimizing Age of Information with a Relay. arXiv.

[24] Amsüss C., Koster M., Li X., Lorenzo C., Pelov A., Ranganathan A., Tschofenig H. (2023). Message Queuing Telemetry Transport (MQTT) and the Constrained Application Protocol (CoAP). RFC 9431. https://datatracker.ietf.org/doc/rfc9431/.

[25] Gao H., Song L., Liu J. EdgeDRNN: Enabling Low-Latency Recurrent Neural Network Edge Inference. Proceedings of the 2020 IEEE International Conference on Artificial Intelligence Circuits and Systems (AICAS).

[26] Shelby Z., Hartke K., Bormann C., Frank B. (2014). The Constrained Application Protocol (CoAP). RFC 7252. https://datatracker.ietf.org/doc/html/rfc7252.

[27] Gibbs M., Kanjo E. Realising the Power of Edge Intelligence: Addressing the Challenges in AI and tinyML Applications for Edge Computing. Proceedings of the 2023 IEEE International Conference on Edge Computing and Communications (EDGE).

[28] Qin Q., Ye H. (2023). Age of Information Joint Optimization for an Energy Harvesting Network with Erasure Channel. IEEE Access.

[29] Cao Q., Yu L., Wang Z., Zhan S., Quan H., Yu Y., Khan Z., Koubaa A. (2021). Wild Animal Information Collection Based on Depthwise Separable Convolution in Software Defined IoT Networks. Electronics.

[30] Simonyan K., Zisserman A. Very Deep Convolutional Networks for Large-Scale Image Recognition. Proceedings of the International Conference on Learning Representations (ICLR).

[31] Zamanidoost Y., Ould-Bachir T., Martel S. (2025). OMS-CNN: Optimized Multi-Scale CNN for Lung Nodule Detection Based on Faster R-CNN. IEEE J. Biomed. Health Inform..

[32] Jeong S., Lee J., Kim K. (2021). Efficient Deep Learning Models for Embedded IoT Systems. J. Syst. Archit..

[33] Zhou C., Li G., Li J., Guo B. (2019). Energy-Aware Real-Time Data Processing for IoT Systems. IEEE Access.

[34] Zhao W.X., Li J., Wang S., Liu Y., Chang B., Li Y., Du J., Wen J.R., Tang J. (2023). A Survey of Large Language Models. IEEE Trans. Neural Netw. Learn. Syst..

[35] Kumar A., Sharma A., Bharti V., Singh A.K., Singh S.K., Saxena S. (2021). MobiHisNet: A Lightweight CNN in Mobile Edge Computing for Histopathological Image Classification. IEEE Internet Things J..

[36] Sanmartín D., Prohaska V. (2021). Exploration of TPUs for AI Applications. IEEE Robot. Autom. Mag..

[37] Sudharsan B. TinyML-CAM: 80 FPS Image Recognition in 1 KB RAM. Proceedings of the 28th Annual International Conference on Mobile Computing and Networking.

[38] Zhang X., Wang Y.-L., Byun H. (2025). Divisive hierarchical clustering for energy saving and latency reduction in UAV-assisted WSANs. EURASIP J. Wirel. Commun. Netw..

[39] Mani S., Kishoreraja P.C., Joseph C., Manoharan R. (2025). Hybrid intrusion detection model for hierarchical wireless sensor network using federated learning. IAES Int. J. Artif. Intell..

[40] 360Connect How Much Does a Business Security System Cost?. https://www.360connect.com/product-blog/business-security-system-cost/.

[41] CCTV Camera World NVR—Network Video Recorder. https://www.cctvcameraworld.com/network-video-recorders-ip-cameras.html.

[42] Safe and Sound Security Business Alarm Systems Pricing: Complete Guide.2024. https://getsafeandsound.com/blog/business-alarm-systems-pricing/.

[43] Shorten C., Khoshgoftaar T.M. (2019). A Survey on Image Data Augmentation for Deep Learning. J. Big Data.

[44] Gan W., Zhao R., Ma Y., Ning X. (2025). TSF-MDD: A Deep Learning Approach for Electroencephalography-Based Diagnosis of Major Depressive Disorder with Temporal–Spatial–Frequency Feature Fusion. Bioengineering.

[45] Sze V., Chen Y., Yang T.J., Emer J.S. (2017). Efficient Processing of Deep Neural Networks: A Tutorial and Survey. Proc. IEEE.

[46] Kumar V.N., Srisuma V., Mubeen S., Mahwish A., Afrin N., Jagannadham D.B.V., Narasimharao J. (2023). Anomaly-based hierarchical intrusion detection for black hole attack detection and prevention in WSN. Lect. Notes Netw. Syst..

[47] Coelho G.A., Jesus T.C., Costa D.G. (2023). Urban emergency detection system using hierarchical, collaborative and configurable wireless sensor networks. 2023 XIII Brazilian Symposium on Computing Systems Engineering (SBESC), Porto Alegre, Brazil, 21–24 November 2023.

[48] Jacob S., Kligys B., Chen B., Zhu M., Tang Y., Howard A., Adam H., Kalenichenko D. Quantization and Training of Neural Networks for Efficient Integer-Arithmetic-Only Inference. Proceedings of the IEEE Conference on Computer Vision and Pattern Recognition (CVPR).

[49] Babu A.R., Basavaraddi C.C.S., S R., Mouleeswaran S.K., Sahoo S.K., Solainayagi P. Cloud-based Data Analytics for Automated Coastal Cleanup Robots with Convolutional Neural Network. Proceedings of the 2024 8th International Conference on I-SMAC (IoT in Social, Mobile, Analytics and Cloud) (I-SMAC).

[50] Wang L., Wei G., Huang K., Lao Y., Liu X., Li Z. Research on Design of Internet of Things Gateway for Adaptive Data Acquisition of Power Transmission, Transformation and Distribution Based on Edge Intelligence. Proceedings of the 2023 Asia-Europe Conference on Electronics, Data Processing and Informatics (ACEDPI).

[51] Baller S.P., Jindal A., Chadha M., Gerndt M. (2021). DeepEdgeBench: Benchmarking Deep Neural Networks on Edge Devices. IEEE Access.

[52] Shahid A., Mushtaq M. A Survey Comparing Specialized Hardware and Evolution in TPUs for Neural Networks. Proceedings of the 2020 IEEE 23rd International Multitopic Conference (INMIC).

[53] Tobiasz R., Wilczyński G., Graszka P., Czechowski N., Łuczak S. (2023). Edge Devices Inference Performance Comparison. IEEE Access.

[54] Mohammadi M., Elbtity M.E., Zand R. Efficient Deployment of Transformer Models on Edge TPU Accelerators: A Real System Evaluation. Proceedings of the 2023 Architecture and System Support for Transformer Models (ASSYST).

[55] Pinzari A., Arafa M.T., Eltawil A.M. (2024). Inside the AI Accelerators: From High Performance to Energy Efficiency. IEEE Trans. Comput..

[56] Chen J., Teo T.H., Kok C.L., Koh Y.Y. (2024). A Novel Single-Word Speech Recognition on Embedded Systems Using a Convolution Neuron Network with Improved Out-of-Distribution Detection. Electronics.

